# High Self-Reported Prevalence of Kidney Stones in Trinidad and Tobago: Results of a Cross-Sectional Online Survey

**DOI:** 10.7759/cureus.57651

**Published:** 2024-04-05

**Authors:** Satyendra A Persaud, Satish Jankie, Roger Andrews, Saleem Varachhia, Michael Morris

**Affiliations:** 1 Faculty of Medical Sciences, University of the West Indies, St Augustine, TTO; 2 Department of Physics, University of the West Indies, St Augustine, TTO

**Keywords:** ureteral calculi, end stage renal disease (esrd), trinidad and tobago, renal calculi (kidney stones), treatment of urolithiasis, urolithiasis epidemiology

## Abstract

Introduction

No data exist on the prevalence of kidney stone disease in Trinidad and Tobago. Local clinicians have noted that the disease is very common, and this study represents the first attempt to investigate the prevalence of urolithiasis in these islands.

Objectives

The objective is to estimate the prevalence of kidney stone disease in Trinidad and Tobago and to investigate the epidemiology of the disease.

Methods

An online survey using the online tool Survey Monkey was distributed among members of the public via instant messaging and social media. The survey captured data relating to the stone status and demographics of respondents.

Results

1225 patients completed the survey of whom 46.5% were males and 53.5% were females. Respondents were equally distributed throughout the country. 16.74% of those surveyed indicated that they were currently affected by stones confirmed by imaging. Kidney stones were more common among Trinidadians of East Indian ancestry (20.6% vs 10.6%). Positive correlations were established between kidney stones and the presence of hypertension, diabetes, and gout. Persons with kidney stones were more likely to have a family member with the disease - 45.6% vs 31.4% among those without kidney stones.

Conclusion

This study demonstrates a high self-reported prevalence of kidney stones in Trinidad and Tobago.

## Introduction

Kidney stones, or renal calculi accrue from agglomeration of crystals made from minerals and salts along the urinary tract. They are diagnosed by clinical symptoms and radiological assessment and may cause pain, hematuria, and urinary tract obstruction [1]. According to Sorokin et al., kidney stone prevalence is highest in North America (7%-13%), followed by Europe (5%-9%) then Asia (1%-5%) [2]. The prevalence may be higher in developed countries due to enhanced detection rates, but also dietary considerations such as higher salt and protein intake. In developing countries, malnutrition and scarce water supplies may be contributing factors to its prevalence [3]. Data across different time periods indicate that there has been a general increase in the prevalence of kidney stones worldwide, creating a greater financial burden on healthcare systems [4].

Data regarding the prevalence of kidney stones in the Caribbean are limited. Anatol found that the most significant increase in kidney stone prevalence globally from 1990 to 2019 occurred in the Caribbean [5]. A small study in Trinidad identified that many kidney stone patients had a family history of the disease as well as a low magnesium intake [6]. Additionally, 61% of patients were of East Indian descent, while the rest were African or mixed race. No study has been done locally to estimate the prevalence of the disease despite the observations by local practitioners that kidney stones are very common. Our work, to the best of our knowledge, represents the first study of this nature to be conducted locally.

## Materials and methods

This was a cross-sectional study and was granted ethical approval from the Ethics Committee of the University of the West Indies, St Augustine Campus (CREC-SA.1748/09/2022).

This study was conducted over 12 weeks using a questionnaire that was developed by the study team based on an extensive literature review with all members of the team reviewing the questions. The group developed and revised individual questions to ensure simplicity within the local setting so that they could be understood by the general population regardless of their socioeconomic and educational background. A pilot study was conducted on 50 randomly selected individuals whom we approached at a shopping mall. Based on the feedback received, the questions were further modified to ensure simplicity, clarity, and understanding.

The questionnaire comprised 26 questions and was divided into two sections. Section A consisted of seven questions on demographics including age, gender, height, weight, ethnicity, educational level, and area of residence. Section B comprised 12 questions on the respondent’s medical and family history and included if the respondent was diagnosed with a non-communicable disease, where most of the respondent’s time is spent (indoors/outdoors), and whether there was a history of kidney stones among immediate family or extended family members.

The questionnaire was distributed electronically using the online tool SurveyMonkey® to members of the public via social media. Links to the survey were distributed online, in non-health-related communities, and in numerous chat groups representative of the Trinidadian population. Inclusion criteria included age >18 and being a resident of Trinidad and Tobago. Data were collected over a three-month period from August to October 2022.

The sample size required for this study was determined to be 384 persons using a margin of error of 5% and a confidence interval of 95%. The study team aimed to attain a larger sample size as it would be more representative of the population. Statistical analysis was conducted using the Statistical Package for the Social Sciences (SPSS®) version 24 (IBM Corp., Armonk, NY). Descriptive statistics were used to summarize the respondent’s demographic variables and Chi-square analysis was used to detect significant associations between demographics and measured variables. A p-value < 0.05 was considered significant.

## Results

A total of 1,225 individuals completed the survey of which 46.5% were males and 53.5% females. Stones were more common among males (19.5%) than females (13.8%) (p=0.007). Overall, 16.74% of respondents reported being currently affected by kidney stones confirmed by imaging, whether symptomatic or asymptomatic. More than half (65%) reported stones on one side and 35% bilateral stones. Overall, 19.2% of respondents spent the majority of their time outdoors: among those without active kidney stones at the time of the study, 33.3% spent most of their time outdoors while only 16.4% of those without stones spent the majority of their time outdoors (p<0.001).

Location

Participants were grouped into five locations. Most patients were from Southern Trinidad (35.6%). The prevalence of stones did not vary by geographic location (p=0.516) (Table 1).

**Table 1:** Influence of geographic location on the presence of kidney stones

Stone status	East (n/%)	North (n/18%)	Central (n/%)	South (n/%)	Tobago (n/%)	Total
Stone formers	45 (18.3)	28 (12.9)	54 (18.1)	73 (16.7)	4 (14.3)	204
Non-stone formers	201(81.7)	189 (87.1)	244 (81.9)	363 (83.3)	24 (85.7)	1021
Total	246	217	298	436	28	1225

Family history

There was a strong association between the presence of kidney stones and a family history of stones (p<0.001). Among all participants, 33.8% reported having a first-degree relative afflicted by stones. However, among respondents currently affected by kidney stones at the time of the study, this number was 45.6%. A history of kidney stones among members of the extended family was also more common among those affected by stones (p=0.029).

Race

Most Trinidadians are either of Afro or East Indian descent. Race was self-identified, and the study population consisted mostly of 616 (50.3%) persons of Indo-Trinidadian descent and 255 (20.8%) persons of Afro-Trinidadian descent with the rest being primary persons of mixed ethnicities. Table 2 illustrates the breakdown of the cohort based on race. There was a strong correlation between race and the presence of kidney stones with prevalence being much higher among Indo-Trinidadians compared to Afro-Trinidadians (20.6% vs 10.6%) (p=0.001).

**Table 2:** Influence of race on the prevalence of kidney stones

Stone status	Afro-Trinidadian (n/%)	Indo-Trinidadian (n/%)	Mixed (n/%)	Other (n/%)	Total
Stone formers	27 (10.6)	127 (20.6)	41 (13.7)	9 (16.7)	204
Non-stone formers	228 (89.4)	489 (79.4)	259 (8.6)	45 (83.3)	1021
Total	255	616	300	54	1225

Comorbidities 

Table 3 illustrates comorbidities among the study cohort. Hypertension was the most common comorbid condition affecting 21.6% of respondents, followed by diabetes in 11.8%. Both conditions were significantly more common among stone formers.

**Table 3:** Prevalence of comorbidities among the study population Statistical significance assessed by chi-squared testing

Comorbidity	Stone formers (n/%)	Non-stone formers (n/%)	Statistical significance
Hypertension	65 (31.9)	199 (19.5)	p<0.01
Diabetes	44 (21.6)	100 (9.8)	P<0.01
Ischaemic heart disease	8 (3.9)	28 (2.7)	P>0.05
Gout	11 (5.4)	22 (2.2)	P<0.05

## Discussion

Our study showed a high self-reported prevalence of kidney stones. This study, to the best of our knowledge, is the first of its kind to attempt to quantify stone disease locally or even within the English-speaking Caribbean. In a population-based study in the United States, a self-reported prevalence of 8.6% was identified [7]. In a recent study based on the National Health and Nutrition Examination Survey (NHANES) data, the self-reported prevalence of stones was 10.1% [8]. In a global study, Romero noted that prevalence rates varied considerably, ranging from 2.62% to 14.8% [9]. Our self-reported prevalence of 16.74% was considerably higher than these values and this is certainly hypothesis-generating. 

In our work, stone formers were more likely to spend time outdoors. Trinidad and Tobago is a tropical country with intense, year-round sunshine and it is possible that this may influence stone prevalence. It is known that seasonal temperature variation and exposure to sunlight also impact the incidence of kidney stones. One worldwide study found that during periods of higher temperature, more kidney stones were detected [10]. This is due to the low urine volume which results from increased fluid loss at higher temperatures. For example, Stamatelou also concluded that populations in southern parts of the United States have a higher kidney stone prevalence than other areas due to their warmer temperatures [11]. Urban centers also show higher temperatures than surrounding rural areas, so these locations may exhibit higher prevalence rates as well [12]. We also noted a significantly higher prevalence of stones among men and this is in keeping with international trends [13].

The population of Trinidad and Tobago consists primarily of persons of Indian and African ancestry with considerable mixing of ethnicities. We noted a significantly higher prevalence of urolithiasis among persons identifying as Indo-Trinidadian and this conforms to what colleagues have anecdotally observed in local practice [14]. This may be multifactorial in origin with a combination of genetics and environmental factors at play. It has been noted that the prevalence of urinary tract stones in India may be as high as 15% [15].

Diet and lifestyle are the main factors that affect the prevalence of kidney stones and therefore the rise in lifestyle diseases in Western countries will result in a corresponding increase in stone prevalence. Lifestyle diseases such as obesity and diabetes are risk factors for kidney stone formation. Antonelli estimated that by 2030, obesity will cause kidney stone prevalence to increase by 0.36% and diabetes will cause it to increase by 0.72% [16]. Both these diseases result in lower urine pH, which contributes to the formation of kidney stones. A diet high in animal proteins also increases the risk for kidney stones - we did not delve into this in this study, but this certainly warrants further investigation especially in the context of apparent ethnic disparities in kidney stone prevalence locally [17].

Our work confirms clear associations between urolithiasis and a number of chronic health conditions - these have been previously described in the literature but only for the first time in our population. We noted positive associations between urolithiasis and the presence of diabetes, hypertension, and gout. A clear association between diabetes and the prevalence of kidney stones has been demonstrated. In a cross-sectional study of three large cohorts, Taylor et al. noted that the relative risk of having kidney stones among patients with diabetes compared to those without was between 1.31 and 1.67 [18]. The risk of developing hypertension was investigated by Rule and colleagues who followed stone formers and controls for several years. They noted a higher risk of hypertension among stone formers with a hazard ratio of 1.5 [19]. The presence of gout also has a well-established association with the presence of kidney stones. Our study therefore confirms that this trend also holds true in our population. While we looked at common conditions or conditions known to be associated with stones in this preliminary study, we have plans for further in-depth work in the future.

Practitioners in Trinidad and Tobago have always noted, albeit anecdotally, that patients with urolithiasis often had a family history of the disease. We have confirmed this relationship in our population. Almost half of stone formers had a first-degree relative with a history of urolithiasis and the presence of stones was also more common among their extended family. In the Health Professionals follow-up study, men with a family history of kidney stones had a relative risk of 2.57 of developing kidney stones [20]. Other authors have also noted that family history may affect the presentation of the disease. Koyuncu and colleagues noted that stone formation may happen earlier and more frequently among patients with a family history of stones [21].

These findings have real-world and practical implications for how we counsel our patients locally. And importantly, this study provides useful data for policymakers responsible for resource allocation. It should also serve as inspiration or the basis for further research on kidney stone disease in Trinidad and Tobago.

Our study was based on the self-reported incidence of stones, and this would have been a limitation. We attempted as best as we could to mitigate this by specifically asking only for image-confirmed disease, but we admittedly have no way of verifying this. An open appeal to the population may have also biased our study toward stone formers who may have been more likely to respond. This study, limitations notwithstanding, provides guidance for policy planners on the extent of kidney stones in Trinidad and Tobago and we feel it is an important starting point for local research.

## Conclusions

This study suggests a high prevalence of kidney stones in Trinidad and Tobago. A family history of kidney stones was also common among persons with kidney stones. Several chronic diseases such as diabetes and hypertension were also more common among stone formers.

## Appendices

                                          Questionnaire

Dear Sir/Madam

Thank you for taking the time to complete this survey. Please answer each question as honestly as possible. This survey is completely anonymous and no personal data will be collected. 

_____________________________________________________________________________________________________________________________

How old are you?   ____

What is your gender?   Male   Female

Where in Trinidad and Tobago are you from?   North  South  East  Central  Tobago

What is your ethnicity?    Indo-Trinidadian    Afro-Trinidadian   Mixed    Other

Do you have stones in your kidneys or ureters (kidney tubes) that have been confirmed with X-Rays, ultrasound or CT scan?    Yes   No

Which of the following best describes your kidney stone history?

I have stones now and in the past

This is my first stone episode

I don’t have stones at present but had them in the past

I have never had kidney stones

If you have/had kidney stones, were they on -   One side      Both sides

Do you have close family members (father, mother, siblings)who have kidney stones?

Yes

No

Do you have extended family members (aunts, uncles, cousins) who have kidney stones?

Yes

No

Do you have any of the following medical conditions?  Heart Disease, Diabetes, Hypertension Gout

Where do you spend the majority of your day?   Indoors   Outdoors

## References

[1] Rule AD, Lieske JC, Pais VM Jr (2020). Management of kidney stones in 2020. JAMA.

[2] Sorokin I, Mamoulakis C, Miyazawa K, Rodgers A, Talati J, Lotan Y (2017). Epidemiology of stone disease across the world. World J Urol.

[3] Stamatelou K, Goldfarb DS (2023). Epidemiology of kidney stones. Healthcare (Basel).

[4] Hyams ES, Matlaga BR (2014). Economic impact of urinary stones. Transl Androl Urol.

[5] Lang J, Narendrula A, El-Zawahry A, Sindhwani P, Ekwenna O (2022). Global trends in incidence and burden of urolithiasis from 1990 to 2019: an analysis of Global Burden of Disease study data. Eur Urol Open Sci.

[6] Anatol T, Pinto Pereira L, Simeon D, Sawh L (2003). Risk factors for urinary tract calculi in Trinidad. Trop Med Int Health.

[7] Forbes CM, Nimmagadda N, Kavoussi NL, Xu Y, Bejan CA, Miller NL, Hsi RS (2023). Kidney stone prevalence based on self-report and electronic health records: insight into the prevalence of active medical care for kidney stones. Urology.

[8] Chewcharat A, Curhan G (2021). Trends in the prevalence of kidney stones in the United States from 2007 to 2016. Urolithiasis.

[9] Romero V, Akpinar H, Assimos DG (2010). Kidney stones: a global picture of prevalence, incidence, and associated risk factors. Rev Urol.

[10] Geraghty RM, Proietti S, Traxer O, Archer M, Somani BK (2017). Worldwide impact of warmer seasons on the incidence of renal colic and kidney stone disease: evidence from a systematic review of literature. J Endourol.

[11] Stamatelou KK, Francis ME, Jones CA, Nyberg LM, Curhan GC (2003). Time trends in reported prevalence of kidney stones in the United States: 1976-1994. Kidney Int.

[12] Goldfarb DS, Hirsch J (2015). Hypothesis: urbanization and exposure to urban heat islands contribute to increasing prevalence of kidney stones. Med Hypotheses.

[13] Edvardsson VO, Indridason OS, Haraldsson G, Kjartansson O, Palsson R (2013). Temporal trends in the incidence of kidney stone disease. Kidney Int.

[14] Kodama H, Ohno Y (1989). Descriptive epidemiology of urolithiasis (Article in Japanese). Hinyokika Kiyo.

[15] Prakash R, Arunachalam N (2019). Prevalence and socio-demographic status on kidney stone patients in Thanjavur district, Tamil Nadu, India. Int J Commun Med Public Health.

[16] Antonelli JA, Maalouf NM, Pearle MS, Lotan Y (2014). Use of the National Health and Nutrition Examination Survey to calculate the impact of obesity and diabetes on cost and prevalence of urolithiasis in 2030. Eur Urol.

[17] Tracy CR, Best S, Bagrodia A (2014). Animal protein and the risk of kidney stones: a comparative metabolic study of animal protein sources. J Urol.

[18] Taylor EN, Stampfer MJ, Curhan GC (2005). Diabetes mellitus and the risk of nephrolithiasis. Kidney Int.

[19] Kittanamongkolchai W, Mara KC, Mehta RA (2017). Risk of hypertension among first-time symptomatic kidney stone formers. Clin J Am Soc Nephrol.

[20] Curhan GC, Willett WC, Rimm EB, Stampfer MJ (1997). Family history and risk of kidney stones. J Am Soc Nephrol.

[21] Koyuncu HH, Yencilek F, Eryildirim B, Sarica K (2010). Family history in stone disease: how important is it for the onset of the disease and the incidence of recurrence?. Urol Res.

